# Image Processing Using FPGAs

**DOI:** 10.3390/jimaging5050053

**Published:** 2019-05-10

**Authors:** Donald G. Bailey

**Affiliations:** Department of Mechanical and Electrical Engineering, School of Food and Advanced Technology, Massey University, Palmerston North 4442, New Zealand; D.G.Bailey@massey.ac.nz

**Keywords:** field programmable gate arrays (FPGA), image processing, hardware/software co-design, memory management, segmentation, image analysis, compression

## Abstract

Nine articles have been published in this Special Issue on image processing using field programmable gate arrays (FPGAs). The papers address a diverse range of topics relating to the application of FPGA technology to accelerate image processing tasks. The range includes: Custom processor design to reduce the programming burden; memory management for full frames, line buffers, and image border management; image segmentation through background modelling, online K-means clustering, and generalised Laplacian of Gaussian filtering; connected components analysis; and visually lossless image compression.

## 1. Introduction to This Special Issue

Field programmable gate arrays (FPGAs) are increasingly being used for the implementation of image processing applications. This is especially the case for real-time embedded applications, where latency and power are important considerations. An FPGA embedded in a smart camera is able to perform much of the image processing directly as the image is streamed from the sensor, with the camera providing a processed output data stream, rather than a sequence of images. The parallelism of hardware is able to exploit the spatial (data level) and temporal (task level) parallelism implicit within many image processing tasks. Unfortunately, simply porting a software algorithm onto an FPGA often gives disappointing results, because many image processing algorithms have been optimised for a serial processor. It is usually necessary to transform the algorithm to efficiently exploit the parallelism and resources available on an FPGA. This can lead to novel algorithms and hardware computational architectures, both at the image processing operation level and also at the application level.

The aim of this Special Issue is to present and highlight novel algorithms, architectures, techniques, and applications of FPGAs for image processing. A total of 20 submissions were received for the Special Issue, with nine papers being selected for final publication.

## 2. Contributions

Programming an FPGA to accelerate complex algorithms is difficult, with one of four approaches commonly used [1]:

- Custom hardware design of the algorithm using a hardware description language, optimised for performance and resources;
- implementing the algorithm by instantiating a set of application-specific intellectual property cores (from a library);
- using high-level synthesis to convert a C-based representation of the algorithm to synthesisable hardware; or
- mapping the algorithm onto a parallel set of programmable soft-core processors.

The article by Siddiqui et al. [1] took this last approach, and describes the design of an efficient 16-bit integer soft-core processor, IPPro, capable of operating at 337 MHz, specifically targetting the dataflow seen in complex image processing algorithms. The presented architecture uses dedicated stream access instructions on the input and output, with a 32-element local memory for storing pixels and intermediate results, and a separate 32-element kernel memory for storing filter coefficients and other parameters and constants. The exploitation of both data-level parallelism and task-level parallelism is demonstrated through the mapping of a K-means clustering algorithm onto the architecture, showing good scalability of processing speed with multiple cores. A second case study of traffic sign recognition is partitioned between the IPPro cores and an ARM processor, with the colour conversion and morphological filtering stages mapped to the IPPro. Again, the use of parallel IPPro cores can significantly accelerate these tasks, compared to conventional software, without having to resort to the tedious effort of custom hardware design.

Garcia et al. [2] worked on the thesis that the image processing operations which require random access to the whole frame (including iterative algorithms) are particularly difficult to realise in FPGAs. They investigate the mapping of a frame buffer onto the memory resources of an FPGA, and explore the optimal mapping onto combinations of configurable on-chip memory blocks. They demonstrate that, for many image sizes, the default mapping by the synthesis tools results in poor utilisation, and is also inefficient in terms of power requirements. A procedure is described that determines the best memory configuration, based on balancing resource utilisation and power requirements. The mapping scheme is demonstrated with optical flow and mean shift tracking algorithms.

On the other hand, local operations (such as filters) only need part of the image to produce an output, and operate efficiently in stream processing mode, using line buffers to cache data for scanning a local window through the image. This works well when the image size is fixed, and is known in advance. Two situations where this approach is less effective [3] are in the region of interest processing, where only a small region of the image is processed (usually determined from the image contents at run-time), and cloud processing of user-uploaded images (which may be of arbitrary size). This is complicated further in high-speed systems, where the real-time requirements demand processing multiple pixels in every clock cycle, because, if the line width is not a multiple of the number of pixels processed each cycle, then it is necessary to assemble the output window pixels from more than one memory block. Shi et al. [3], in their paper, extend their earlier work on assembling the output window to allow arbitrary image widths. The resulting line buffer must be configurable at run-time, which is achieved through a series of “instructions”, which control the assembly of the output processing window when the required data spans two memory blocks. Re-configuration only takes a few clock cycles (to load the instructions), rather than conventional approach of reconfiguring the FPGA each time the image width changes. The results demonstrate better resource utilisation, higher throughput, and lower power than their earlier approach.

When applying window operations to an image, the size of the output image is smaller than the input because data is not valid when the window extends beyond the image border. If necessary, this may be mitigated by extending the input image to provide data to allow such border pixels to be calculated. Prior work only considered border management using direct form filter structures, because the window formation and filter function can be kept independent. However, in some applications, transpose-form filter structures are desirable because the corresponding filter function is automatically pipelined, leading to fewer resources and faster clock frequencies. Bailey and Ambikumar [4] provide a design methodology for border management using transpose filter structures, and show that the resource requirements are similar to those for direct-form border management.

An important task in computer vision is segmenting objects from a complex background. While there are many background modelling algorithms, the complexity of robust algorithms make them difficult to realise on an FPGA, especially for larger image sizes. Chen et al. [5] address scalability issues with increasing image size by using super-pixels—small blocks of adjacent pixels that are treated as a single unit. As each super-pixel is considered to be either object or background, this means that fewer models need to be maintained (less memory) and fewer elements need to be classified (reduced computation time). Using hardware/software co-design, they accelerated the computationally expensive steps of Gaussian filtering and calculating the mean and variance within each super-pixel with hardware, with the rest of the algorithm being realised on the on-chip CPU. The resulting system gave close to state-of-the-art classification accuracy.

A related paper, by Badawi and Bilal [6], used K-means clustering to segment objects within video sequences. Rather than taking the conventional iterative approach to K-means clustering, they rely on the temporal coherence of video streams and use the cluster centres from the previous frame as initialisation for the current frame. Additionally, rather than waiting until the complete frame has been accumulated before updating the cluster centres, an online algorithm is used, with the clusters updated for each pixel. To reduce the computational requirements, the centres are updated using a weighted average. They demonstrate that, for typical video streams, this gives similar performance to conventional K-means algorithms, but with far less computation and power.

In another segmentation paper, Zhou et al. [7] describe the use of a generalised Laplacian of Gaussian (LoG) filter for detecting cell nuclei for a histopathology application. The LoG filters detect elliptical blobs at a range of scales and orientations. Local maxima of the responses are used as candidate seeds for cell centres, and mean-shift clustering is used to combine multiple detections from different scales and orientations. Their FPGA design gave modest acceleration over a software implementation on a high-end computer.

Given a segmented image, a common task is to measure feature vectors of each connected component for analysis. Bailey and Klaiber [8] present a new single-pass connected components analysis algorithm, which does this with minimum latency and relatively few resources. The key novelty of this paper is the use of a zig-zag based scan, rather than a conventional raster scan. This eliminates the end-of-row processing for label resolution by integrating it directly within the reverse scan. The result is true single-pixel-per-clock-cycle processing, with no overheads at the end of each row or frame.

An important real-time application of image processing is embedded online image compression for reducing the data bandwidth for image transmission. In the final paper within this Special Issue, Wang et al. [9] defined a new image compression codec which works efficiently with a streamed image, and minimises the perceptual distortion within the reconstructed images. Through small local filters, each pixel is classified as either an edge, a smooth region, or a textured region. These relate to a perceptual model of contrast masking, allowing just noticeable distortion (JND) thresholds to be defined. The image is compressed by downsampling; however, if the error in any of the contributing pixels exceeds the visibility thresholds, the 2 × 2 block is considered a region of interest, with the 4 pixels coded separately. In both cases, the pixel values are predicted using a 2-dimensional predictor, and the prediction residuals are quantised and entropy-encoded. Results typically give a visually lossless 4:1 compression, which is significantly better than other visually lossless codecs.

## 3. Conclusions

Overall, this collection of papers reflects the diversity of approaches taken to applying FPGAs to image processing applications. From one end, using the programmable logic to design lightweight custom processors to enable parallelism, through overcoming some of the limitations of current high-level synthesis tools, to the other end with the design of custom hardware designs at the register-transfer level.

The range of image processing techniques include filtering, segmentation, clustering, and compression. Applications include traffic sign recognition for autonomous driving, histopathology, and video compression.

## References

[1] Siddiqui F., Amiri S., Minhas U.I., Deng T., Woods R., Rafferty K., Crookes D. (2019). FPGA-based processor acceleration for image processing applications. J. Imaging.

[2] Garcia P., Bhowmik D., Stewart R., Michaelson G., Wallace A. (2019). Optimized memory allocation and power minimization for FPGA-based image processing. J. Imaging.

[3] Shi R., Wong J.S., So H.K.H. (2019). High-throughput line buffer microarchitecture for arbitrary sized streaming image processing. J. Imaging.

[4] Bailey D.G., Ambikumar A.S. (2018). Border handling for 2D transpose filter structures on an FPGA. J. Imaging.

[5] Chen A.T.Y., Gupta R., Borzenko A., Wang K.I.K., Biglari-Abhari M. (2018). Accelerating SuperBE with hardware/software co-design. J. Imaging.

[6] Badawi A., Bilal M. (2019). High-level synthesis of online K-Means clustering hardware for a real-time image processing pipeline. J. Imaging.

[7] Zhou H., Machupalli R., Mandal M. (2019). Efficient FPGA implementation of automatic nuclei detection in histopathology images. J. Imaging.

[8] Bailey D.G., Klaiber M.J. (2019). Zig-zag based single pass connected components analysis. J. Imaging.

[9] Wang Z., Tran T.H., Muthappa P.K., Simon S. (2019). A JND-based pixel-domain algorithm and hardware architecture for perceptual image coding. J. Imaging.

